# Association of benign gynaecological diseases and risk of endometrial and ovarian cancers

**DOI:** 10.7150/jca.39626

**Published:** 2020-03-04

**Authors:** Fanghua Shen, Yang Liu, Luling Lin, Min Zhao, Qi Chen

**Affiliations:** 1 Suzhou Ninth People's Hospital, Suzhou, Jiangsu Province, China.; 2 Nanjing Medical University, Nanjing, China.; 3 Liggins Institute, The University of Auckland, New Zealand.; 4 Department of Gynaecology, The affiliated Wuxi Maternity and Child Health Care Hospital of Nanjing Medical University, China.; 5 The Hospital of Obstetrics & Gynaecology, Fudan University, China.; 6 Department of Obstetrics & Gynaecology, The University of Auckland, New Zealand.

**Keywords:** uterine fibroids, endometriosis, adenomyosis, endometrial cancer, ovarian cancer

## Abstract

**Objective**: Gynaecologic benign diseases such uterine fibroids share similar pathogeneses with endometrial and ovarian cancers. Whether a history of uterine fibroids increases the risk of developing endometrial or ovarian cancers is controversial, due to uterine fibroids was self-reported in those studies.

**Methods**: In our current case-control study, 268 women with endometrial cancer and 108 women with ovarian cancer were included. In addition, 500 women without gynaecological cancers were randomly selected as a control group. Uterine fibroids in both groups were clinically diagnosed by pelvic examination and ultrasound. Data on age, parity, gravida, stages of cancers and history of uterine fibroids, endometriosis and adenomyosis were collected from hospital database.

**Results**: After adjusted age and parity, the odds of women with history of uterine fibroids or endometriosis were lower in women with endometrial cancer than controls (odds ratio: 0.148, 95% CI: 0.097, 0.225, or 0.360, 95% CI: 0094, 1.381, respectively). The odds of women with a history of uterine fibroids or endometriosis were lower in women with ovarian cancer than controls (odds ratio: 0.141, 95% CI: 0.085, 0.235, or 1.057, 95% CI: 0.377, 2.963, respectively). However, the odds of women with a history of adenomyosis were higher in women with endometrial or ovarian cancers than controls (odd ratio: 3.757, 95% CI: 1.858, 7.599 or 2.341, 95% CI: 1.086, 5.045, respectively).

**Conclusion**: Our observational data suggested that uterine fibroids or endometriosis may be not associated with the increased risk of developing endometrial or ovarian cancer. However, a history of adenomyosis may do.

## Introduction

Endometrial and ovarian cancers are the sixth and seventh-most common cancers in women, respectively (World Cancer Report 2014), and the incidence of endometrial and ovarian cancers has been significantly increasing across all ethnicities worldwide since last decade 1,2. Although the underlying mechanism of endometrial and ovarian cancers are still not fully understood, most risk factors for endometrial cancer involve high levels of estrogens and an estimated 40% of endometrial cancer are thought to be associated with obesity (World Cancer Report 2014). In addition, the Million Women Study also suggested that estrogen replacement therapy for post-menopausal women can increase their risk of developing ovarian cancer. These studies suggested that increased estrogen levels are one of the main risk factors for developing endometrial and ovarian cancers.

Uterine fibroids are non-cancerous growths of the uterus that often appear at reproductive age. It has been reported that 20% to 80% of women develop uterine fibroids by the age of 50 3-6. However, approximately 50% of women with uterine fibroids have no clinical symptoms, such as abdominal pain, anemia and increased menstrual bleeding 4,7. Uterine fibroids are dependent on estrogen and progesterone to grow 8-10. A study has reported that the risk of obese women developing uterine fibroids is 2-3 times greater than women with normal body mass indices 10. Endometriosis and adenomyosis are another two common gynaecologic benign diseases and affect 7% to 15% and 5% to 70% of all women at reproductive age, respectively 11,12. Many cases of endometriosis and adenomyosis were asymptomatic 13. Prolonged exposure of estrogen is thought to be a risk factor for endometriosis 14.

It has been reported that uterine fibroids or endometriosis shares some common pathogeneses with endometrial or ovarian cancer, including some clinical characteristics and symptoms 15. Consequently, this commonality suggests that there is an association between history of uterine fibroids or endometriosis and the development of endometrial or ovarian cancers. Studies have suggested that uterine fibroids and/or endometriosis increased the risk of developing both endometrial and ovarian cancers 16-20. However, another study reported that uterine fibroids were not associated with an increased risk of endometrial cancer (http://www.mayoclinic.org/diseases-conditions/uterine-fibroids/symptoms-causes/dxc-20212514/).

The incidences of endometrial and ovarian cancers vary by ethnicity, with the Asian population having lower incidences of endometrial and ovarian cancers 21. The difference in the incidence of endometrial and ovarian cancers may result in differences in clinical characteristics and pathological parameters by ethnicities. Our previous studies reported that endometrial and ovarian cancers occur more frequently in Chinese women before menopause 22-24, whilst 75% of endometrial and ovarian cancers occur after menopause in Caucasians 25. Our recent unpublished data also showed differences in clinical characteristics and pathological parameters in breast cancer between Chinese and New Zealand Europeans.

Given the differences in clinical and pathological parameters in endometrial and ovarian cancers by ethnicity, we hypothesise that the association of uterine fibroids or endometriosis with endometrial or ovarian cancers may vary between Chinese women and Caucasians. Therefore, we undertook this study to investigate whether uterine fibroids, endometriosis or adenomyosis increases the risk of developing endometrial and ovarian cancers in the Chinese population.

## Methods

This study was approved by the Ethics Committee of Women Maternity and Child Health Hospital, Nanjing Medical University, China. We performed a case-control analysis.

### Study populations

A total of 268 women who were diagnosed with endometrial cancer and 108 women who were diagnosed with ovarian cancer from January 2013 to December 2016 from Wuxi Maternity and Child Health Hospital, Nanjing Medical University, China were all included. In addition, during the study period, 4370 women without gynaecological cancers were admitted to inpatient clinics in our hospital, out of which 500 patients were randomly selected as a control group by using Microsoft Excel (2013 version).

Data on age at diagnosis of endometrial or ovarian cancers, stage of endometrial or ovarian cancers, status of menopause, history of hypertension, diabetes and history of uterine fibroids or endometriosis or adenomyosis were collected from the hospital's electronic database. Parity and gravida were also collected. Uterine fibroids in both groups were clinically diagnosed by gynaecological examination and ultrasound after admission to the hospital.

Endometriosis is diagnosed by physical pelvic examination and transvaginal ultrasound or magnetic resonance imaging (MRI) and is confirmed by histology with the presence of endometrioid glands and stroma, in addition to the clinical signs and symptoms. Adenomyosis is confirmed by histology, when the presence of endometrial tissue more than 2.5 mm below the endomyometrial junction or a JZ thickness of more than 12 mm according to the previous study 26, in addition to physical examination, transvaginal ultrasound or magnetic resonance imaging (MRI).

### Data analysis and statistical analysis

Age at diagnosis was expressed as median and range. We used conditional logistic regression to estimate the odds ratio (OR) 95% confidence intervals (CI) for the association of uterine fibroids, or endometriosis or adenomyosis and risk of developing endometrial or ovarian cancer. We adjusted age at diagnosis and parity for all models. Statistical analyses were performed using SAS (v.9.4, SAS Institute, Cary, NC, USA) and a two-tailed type 1 error rate of 0.05 as the threshold for statistical significance.

## Results

### General characteristics of the study population

The general characteristics of study participants are summarised in Table 1. The median age was 54 (range 29-85) years in women with endometrial cancer, 50 (range 19-74) years in women with ovarian cancer and 41 (range 19-85) years in women in the control group. The age in controls were significantly younger than either women with endometrial or ovarian cancer (p<0.0001). There were less menopausal women in controls group, compared to endometrial cancer (12.8% vs 61%, p<0.0001) or ovarian cancer (12.8% vs 37%, P<0.0001). More women in endometrial or ovarian cancer group had live births, compared to controls (97% vs 79%, p<0.0001, or 89% vs 79%, p=0.0037). More women in endometrial cancer group had a history of pregnancy, compared to controls (97% vs 85%, p<0.0001). While there was no difference in gradvida between women with ovarian cancer and controls (90% vs 85%, p=0.0324).

### Association of uterine fibroids or adenomyosis or endometriosis and endometrial cancer

Of 268 women with endometrial cancer, there were 61 (23%) cases with history of uterine fibroids, 30 (12%) cases with history of adenomyosis and 4 (1.5%) cases with history of endometriosis (Table 2). While in controls, there were 320 (64%) cases with history of uterine fibroids, 23 (4%) cases with history of adenomyosis and 23 (4%) cases with history of endometriosis (Table 2). Because age and parity are associated with developing endometrial cancer, we then adjusted these factors for further analysis. The adjusted odds ratio of women with history of uterine fibroids developing endometrial cancer was 0.148 (95% CI: 0.097, 0.225, p<0.0001). The adjusted odds ratio of women with history of adenomyosis developing endometrial cancer was 3.757 (95% CI: 1.858, 7.599, p=0.0002). The adjusted odds ratio of women with history of endometriosis developing endometrial cancer was 0.360 (95% CI: 0094, 1.381, p=0.1362).

### Association of uterine fibroids or adenomyosis or endometriosis and ovarian cancer

Of 108 women with ovarian cancer, there were 23 (21%) cases with a history of uterine fibroids, 12 (11%) cases with a history of adenomyosis and 5 (4.6%) cases with a history of endometriosis (Table 3). Because age and parity are associated with developing ovarian cancer, we then adjusted these factors for further analysis. The adjusted odds ratio of women with a history of uterine fibroids developing ovarian cancer was 0.141 (95% CI: 0.085, 0.235, p<0.0001). The adjusted odds ratio of women with a history of adenomyosis developing ovarian cancer was 2.341 (95% CI: 1.086, 5.045, p=0.0299). The adjusted odds ratio of women with a history of endometriosis developing ovarian cancer was 1.057 (95% CI: 0.377, 2.963, p=0.9162).

## Discussion

In this case-control study with a Chinese population, we found that a history of uterine fibroids or endometriosis was not associated with an increased risk of developing endometrial cancer or ovarian cancer. However, a history of adenomyosis was associated with an increased risk of developing both endometrial and ovarian cancers.

Uterine fibroids, endometriosis and adenomyosis are the most common gynaecological diseases in women at reproductive age. The prevalence of uterine fibroids varied widely from 20% to 80%, regions and ethnicity dependent 3-6. Recently there is a trend toward non-surgical management of uterine fibroids, providing an alternative to hysterectomy worldwide. This could result in a clinical challenge: how to assess whether uterine fibroids predisposes women to develop endometrial cancer later on in life. It is well known that uterine fibroids shares common pathogenesis, some clinical characteristics and pathological parameters with endometrial cancer. Few studies reported that uterine fibroids increased the risk of developing both endometrial and ovarian cancers 16-20. However, another study reported that uterine fibroids were not associated with an increased risk of developing endometrial cancer (http://www.mayoclinic.org/diseases-conditions/uterine-fibroids/symptoms-causes/dxc-20212514/). In our current study, we also found that uterine fibroids did not increase the risk of developing endometrial cancer. The odds of women with a history of uterine fibroids were lower among women with endometrial cancer than among controls (adjusted odds ratio 0.148, 95% CI: 0.097, 0.225). This finding may indicate that a history of uterine fibroids was a protective factor for developing endometrial cancer in our study population. The lack of a control group in some case-control studies could contribute to the controversial findings in the literature. In those case-control studies 16-18, a history of uterine fibroids in cases and controls were self-reported in questionnaires, which could result in a recall bias. This is because up to 50% of women with uterine fibroids have no clinical symptoms 4,7 and the prevalence of uterine fibroids is higher in the general population when pelvic examination and ultrasound are included (reviewed in 6). Importantly, in Wise's, Fortuny's or Brinton's study 16-18, the prevalence of uterine fibroids in controls was 37% in black women, 20% in white women, and 2.2% in Danes (Table 4). The prevalence of uterine fibroids reported in those studies were significantly lower than what other studies reported in the literature, where the estimated cumulative incidence of uterine fibroids by age 50 years was 80% for black women and 70% for white women 4. The lower prevalence of uterine fibroids in controls reported in those studies could be explained if a large number of those women were not clinically diagnosed with uterine fibroids. In our current study, uterine fibroids in both groups were confirmed by pelvic examination and ultrasound, when the women were admitted to hospital. The prevalence of uterine fibroids in our control cohort was 64%, which is similar to the prevalence of uterine fibroids in the general population as reported in the literature. To better understand the association between uterine fibroids and the risk of developing endometrial cancer, further study is needed.

The association of uterine fibroids and ovarian cancer has rarely been reported 16. In our current study, we found that uterine fibroids was not associated with increased risk of developing ovarian cancer, as opposed to Brinton's study which reported that uterine fibroids increased the risk of developing ovarian cancer 16. As we discussed above, the controversial results between the two studies could be because of the self-reporting of uterine fibroids in Brinton's study, while in our current study, uterine fibroids was diagnosed by pelvic examination and ultrasound after women were admitted to hospital.

In addition to uterine fibroids, because endometriosis also shares common pathogenesis with endometrial or ovarian cancer 15, some studies suggested that endometriosis could increase the risk for developing endometrial and ovarian cancer 16,17,27. This association was strongly related to the subtypes of ovarian cancer 28. However, in our current study, we found that a history of endometriosis was not associated with an increased risk for developing either endometrial or ovarian cancer. The clinical symptoms of endometriosis can be non-discriminatory, and the initial indication of this disease is normally based on a constellation of symptoms such as dysmenorrhea and chronic pelvic pain 13. This can result in a missed diagnosis or false diagnosis of endometriosis 29. Therefore, recall bias can be a limitation of all these studies.

Adenomyosis is another common gynaecological disease in women at reproductive age. Although adenomyosis is generally considered a benign condition with no increased risk for developing cancer, the endometrial tissue within the myometrium could develop endometrioid adenocarcinoma, with potentially deep myometrial invasion 30. Studies have reported that a history of adenomyosis significantly increased the risk of developing endometrial cancer 27, 31-33. In our current study, we also found the odds of women with a history of adenomyosis were around 4 times higher among women with endometrial cancer than the controls. A history with adenomyosis may be a risk factor of developing endometrial cancer (Adjusted odds ratio 3.757; 95% CI: 1.858, 7.599). However, the association of adenomyosis and ovarian cancer has not been fully investigated. A recent study reported that adenomyosis did not increase the risk of developing ovarian cancer 33. Opposed to that study 33, we found that adenomyosis also significantly increased the risk of developing ovarian cancer. After adjusting for age and parity, the odds of women with a history of adenomyosis were around 2.3 times higher in women with ovarian cancer than the controls (adjusted odds ratio 2.341; 95% CI: 1.086, 5.045). However, in Yeh's study 33, parity was not adjusted. It is well known that parity is significantly associated with the development of ovarian cancer. Adenomyosis is characterized by the abnormal presence of endometrial tissue (glands and stroma) within the myometrium with a maximum junctional zone thickness of 12mm (reviewed in 34). As the condition of adenomyosis is estrogen-dependent, it can cause menstrual disorder. It is well known that high levels of estrogen and menstrual disorder are associated with the risk of developing endometrial and ovarian cancers. Taken together our data suggest that a history of adenomyosis could potentially influence the prognosis of the development of endometrial and ovarian cancers.

There are some limitations in our current study. Firstly, this study was done in a single university teaching hospital. The regional difference may result in a bias. Secondly, due to our sample size of women with cancer, we were not able to analyse the association of adenomyosis with different subtypes of ovarian cancer. Thirdly, due to the unavailability of data, we were not able to analyse the association between time of adenomyosis diagnosis and ovarian cancer diagnosis. This association needs to be investigated in the future. In addition, the underlying mechanism of adenomyosis increasing the risk of development endometrial and ovarian cancer is required for future investigation.

In conclusion, in this study with a relatively large sample size, we found that a history of uterine fibroids or endometriosis may not be associated with developing endometrial cancer or ovarian cancer. However, a history of adenomyosis significantly may associate with increased risk of developing both endometrial and ovarian cancers.

## Figures and Tables

**Table 1:** Clinical characteristics of study population

	Endometrial cancer (n=268)	Ovarian cancer (n=108)	Controls (n=500)	P value (Chi square)
Age at diagnosis (years, median/range)	54 (29-85)	50 (19-74)	41 (19-85)	P<0.00001
Menopause Status at diagnosis (number, %)	165 (61%)	43 (37%)	64 (12.8%)	P<0.00001
Parity (number, %)				
P=0	9 (3%)	12 (11%)	106 (21%)	P<0.00001
P=1	153 (57%)	67 (62%)	314 (63%)	
P≥2	106 (40%)	29 (27%)	82 (16%)	
Gravida (number, %)				
G=0	7 (3%)	11 (10%)	76 (15%)	P<0.00001
G=1	31 (11%)	23 (22%)	88 (18%)	
G≥2	230 (86%)	74 (68%)	335 (67%)	
Stage (number, %)				
Ia or IB	212 (79%)	58	N/A	N/A
IIa or IIB	20 (7.5%)	22		
III	33 (12.4%)	20		
IV	3 (0.1%)	8		

**Table 2:** Association of uterine fibroids, endometriosis and adenomyosis and endometrial cancer

	Endometrial cancer (n=268)	Controls (n=500)	Odds Ratio (95% CI)*
Uterine fibroids	61 (23%)	320 (64%)	0.148 (0.097, 0.225)
Adenomyosis	30 (12%)	23 (4%)	3.757 (1.858, 7.599)
Endometriosis	4 (1.5%)	23 (4%)	0.360 (0.094, 1.381)

*Odds ratio was adjusted by age at diagnosis and parity; CI: confidence intervals.

**Table 3:** Association of uterine fibroids, endometriosis and adenomyosis and ovarian cancer

	Ovarian cancer (n=108)	Controls (n=500)	Odds Ratio (95% CI)*
Uterine fibroids	23 (21%)	320 (64%)	0.141 (0.085, 0.235)
Adenomyosis	12 (11%)	23 (4%)	2.341 (1.086, 5.045)
Endometriosis	5 (4.6%)	23 (4%)	1.057 (0.377, 2.963)

*Odds ratio was adjusted by age at diagnosis and parity; CI: confidence intervals.

**Table 4:** Comparison of prevalence of uterine fibroids between our study and other studies reported in the literature

	Ethnicity	Percentage of uterine fibroids in women with endometrial cancer (%, lower CI; upper CI)	Percentage of uterine fibroids in women without endometrial cancer (%, lower CI; upper CI)
Brinton's study 16	Danes	7.2% (5.92%; 8.71%)	2.2% (2.01%, 2.19%)
Fortuny's Study 17	White women (85%)	29% (24.83%; 33.25%)	20% (16.36%; 23.64%)
Wise's study 18	Black women	53% (47.51%; 59.92%)	37% (37.33%; 37.56%)
Our study	Chinese women	22.7% (17.8%; 28.25%)	64% (59.79%; 68.21%)

## References

[1] Cote ML, Ruterbusch JJ, Olson SH, Lu K, Ali-Fehmi R (2015). The Growing Burden of Endometrial Cancer: A Major Racial Disparity Affecting Black Women. Cancer Epidemiol Biomarkers Prev.

[2] Li X, Zheng S, Chen S, Qin F, Lau S, Chen Q (2015). Trends in gynaecological cancers in the largest obstetrics and gynaecology hospital in China from 2003 to 2013. Tumour Biol.

[3] Parazzini F, Di Martino M, Candiani M, Vigano P (2015). Dietary components and uterine leiomyomas: a review of published data. Nutr Cancer.

[4] Baird DD, Dunson DB, Hill MC, Cousins D, Schectman JM (2003). High cumulative incidence of uterine leiomyoma in black and white women: ultrasound evidence. Am J Obstet Gynecol.

[5] Buttram VC Jr, Reiter RC (1981). Uterine leiomyomata: etiology, symptomatology, and management. Fertil Steril.

[6] Stewart EA, Cookson CL, Gandolfo RA, Schulze-Rath R (2017). Epidemiology of uterine fibroids: a systematic review. BJOG.

[7] Moroni RM, Vieira CS, Ferriani RA, Reis RM, Nogueira AA, Brito LG (2015). Presentation and treatment of uterine leiomyoma in adolescence: a systematic review. BMC Womens Health.

[8] Shikora SA, Niloff JM, Bistrian BR, Forse RA, Blackburn GL (1991). Relationship between obesity and uterine leiomyomata. Nutrition.

[9] Sommer EM, Balkwill A, Reeves G, Green J, Beral DV, Coffey K, Million Women Study C (2015). Effects of obesity and hormone therapy on surgically-confirmed fibroids in postmenopausal women. European journal of epidemiology.

[10] Yang Y, He Y, Zeng Q, Li S (2014). Association of body size and body fat distribution with uterine fibroids among Chinese women. Journal of women's health.

[11] Varma R, Rollason T, Gupta JK, Maher ER (2004). Endometriosis and the neoplastic process. Reproduction.

[12] Taran FA, Stewart EA, Brucker S (2013). Adenomyosis: Epidemiology, Risk Factors, Clinical Phenotype and Surgical and Interventional Alternatives to Hysterectomy. Geburtshilfe und Frauenheilkunde.

[13] Sinaii N, Plumb K, Cotton L, Lambert A, Kennedy S, Zondervan K, Stratton P (2008). Differences in characteristics among 1,000 women with endometriosis based on extent of disease. Fertil Steril.

[14] Giudice LC (2010). Clinical practice. Endometriosis. The New England journal of medicine.

[15] Ness RB (2003). Endometriosis and ovarian cancer: thoughts on shared pathophysiology. Am J Obstet Gynecol.

[16] Brinton LA, Sakoda LC, Sherman ME, Frederiksen K, Kjaer SK, Graubard BI, Olsen JH, Mellemkjaer L (2005). Relationship of benign gynecologic diseases to subsequent risk of ovarian and uterine tumors. Cancer Epidemiol Biomarkers Prev.

[17] Fortuny J, Sima C, Bayuga S, Wilcox H, Pulick K, Faulkner S, Zauber AG, Olson SH (2009). Risk of endometrial cancer in relation to medical conditions and medication use. Cancer Epidemiol Biomarkers Prev.

[18] Wise LA, Sponholtz TR, Rosenberg L, Adams-Campbell LL, Kuohung W, LaValley MP, Palmer JR (2016). History of uterine leiomyoma and risk of endometrial cancer in black women. Cancer causes & control: CCC.

[19] DePriest PD, Banks ER, Powell DE, van Nagell JR Jr, Gallion HH, Puls LE, Hunter JE, Kryscio RJ, Royalty MB (1992). Endometrioid carcinoma of the ovary and endometriosis: the association in postmenopausal women. Gynecol Oncol.

[20] Ness RB, Grisso JA, Cottreau C, Klapper J, Vergona R, Wheeler JE, Morgan M, Schlesselman JJ (2000). Factors related to inflammation of the ovarian epithelium and risk of ovarian cancer. Epidemiology.

[21] Park HK, Ruterbusch JJ, Cote ML (2017). Recent Trends in Ovarian Cancer Incidence and Relative Survival in the United States by Race/Ethnicity and Histologic Subtypes. Cancer Epidemiol Biomarkers Prev.

[22] Gao Y, Zhao M, Dai X, Tong M, Wei J, Chen Q (2016). The prevalence of endometrial cancer in pre- and postmenopausal Chinese women. Menopause.

[23] Wan J, Gao Y, Zeng K, Yin Y, Zhao M, Wei J, Chen Q (2016). The levels of the sex hormones are not different between type 1 and type 2 endometrial cancer. Sci Rep.

[24] Chen S, Dai X, Gao Y, Shen F, Ding J, Chen Q (2017). The positivity of estrogen receptor and progesterone receptor may not be associated with metastasis and recurrence in epithelial ovarian cancer. Sci Rep.

[25] Kong A, Johnson N, Kitchener HC, Lawrie TA (2012). Adjuvant radiotherapy for stage I endometrial cancer.

[26] Benagiano G, Brosens I, Habiba M (2014). Structural and molecular features of the endomyometrium in endometriosis and adenomyosis. Hum Reprod Update.

[27] Kok VC, Tsai HJ, Su CF, Lee CK (2015). The Risks for Ovarian, Endometrial, Breast, Colorectal, and Other Cancers in Women With Newly Diagnosed Endometriosis or Adenomyosis: A Population-Based Study. Int J Gynecol Cancer.

[28] Van Gorp T, Amant F, Neven P, Vergote I, Moerman P (2004). Endometriosis and the development of malignant tumours of the pelvis. A review of literature. Best Pract Res Clin Obstet Gynaecol.

[29] Nnoaham KE, Hummelshoj L, Webster P, d'Hooghe T, de Cicco Nardone F, de Cicco Nardone C, Jenkinson C, Kennedy SH, Zondervan KT (2011). Impact of endometriosis on quality of life and work productivity: a multicenter study across ten countries. Fertil Steril.

[30] Ismiil N, Rasty G, Ghorab Z, Nofech-Mozes S, Bernardini M, Ackerman I, Thomas G, Covens A, Khalifa MA (2007). Adenomyosis involved by endometrial adenocarcinoma is a significant risk factor for deep myometrial invasion. Annals of diagnostic pathology.

[31] Taneichi A, Fujiwara H, Takahashi Y, Takei Y, Machida S, Saga Y, Takahashi S, Suzuki M (2014). Influences of uterine adenomyosis on muscle invasion and prognosis of endometrioid adenocarcinoma. Int J Gynecol Cancer.

[32] Matsuo K, Moeini A, Machida H, Scannell CA, Casabar JK, Kakuda M, Adachi S, Garcia-Sayre J, Ueda Y, Roman LD (2016). Tumor Characteristics and Survival Outcome of Endometrial Cancer Arising in Adenomyosis: An Exploratory Analysis. Ann Surg Oncol.

[33] Yeh CC, Su FH, Tzeng CR, Muo CH, Wang WC (2018). Women with adenomyosis are at higher risks of endometrial and thyroid cancers: A population-based historical cohort study. PLoS One.

[34] Agostinho L, Cruz R, Osório F, Alves J, Setúbal A, Guerra A (2017). MRI for adenomyosis: a pictorial review. Insights Imaging.

